# Hidden diversity of the most basal tapeworms (Cestoda, Gyrocotylidea), the enigmatic parasites of holocephalans (Chimaeriformes)

**DOI:** 10.1038/s41598-021-84613-y

**Published:** 2021-03-09

**Authors:** Daniel Barčák, Chia-Kwung Fan, Pasaikou Sonko, Roman Kuchta, Tomáš Scholz, Martina Orosová, Hsuan-Wien Chen, Mikuláš Oros

**Affiliations:** 1 Institute of Parasitology, Slovak Academy of Sciences, Košice, Slovak Republic; 2 Department of Molecular Parasitology and Tropical Diseases, School of Medicine, College of Medicine, Taipei Medical University, Taipei, Taiwan; 3 Department of International PhD Program in Medicine, College of Medicine, Taipei Medical University, Taipei, Taiwan; 4 Institute of Parasitology, Biology Centre of the Czech Academy of Sciences, České Budějovice, Czech Republic; 5 Department of Biological Resources, National Chiayi University, Chiayi City, Taiwan

**Keywords:** Zoology, Ecology

## Abstract

Gyrocotylideans are evolutionary ancient parasitic flatworms, and like their hosts—a relict group of holocephalan fishes (Chimaeriformes)—they are considered to be “living fossils” of a vanished past. However, the species diversity, host associations and biogeography of these most basal tapeworms are poorly known. Herein, we provide evidence of a conspicuous contrast between the genetic and morphological data based on an examination of newly collected and properly processed *Gyrocotyle* specimens (hologenophores) isolated from holocephalans off Taiwan and Argentina. Our molecular data, inferred from three genes (*COI, 28S rRNA, 18S rRNA*), showed unexpected genetic interrelationships among isolates of the genus *Gyrocotyle*, because each of the four genotypes from Taiwan clustered with isolates of distinct gyrocotylideans from the North Atlantic. Three genotypes of *Gyrocotyle* from Taiwan were morphologically almost indistinguishable from each other but represented distinct genetic lineages; a single specimen of *Gyrocotyle* sp. genotype 4 exhibited a clear genetic and morphological distinctness, though its formal description as a new species would be premature. Additionally, specimens of *Gyrocotyle rugosa* Diesing, 1850, from the type host *Callorhinchus callorynchus* from Argentina, provided the first genetic data on the type species of the genus and enabled us to characterise it, which is necessary for future taxonomic studies. The finding of some specimens of *Gyrocotyle* sp. genotype 3 in *Chimaera phantasma*, and another one in *C.* cf. *argiloba*, together with the putative conspecificity of an unidentified gyrocotylidean from *Callorhinchus milii* off Australia and *G. rugosa* from *C. callorynchus* off Argentina, represent evidence that one gyrocotylidean species may parasitise more than one holocephalan host species. Existing taxonomic problems and conflicts between morphological and molecular data on species of *Gyrocotyle* can only be resolved if hologenophores from type hosts and localities of nominal taxa are properly characterised genetically and morphologically.

## Introduction

The order Gyrocotylidea Poche, 1926, represents an enigmatic and the most basal extant group of tapeworms, with a unique monozoic body and a posteriorly located funnel-shaped adhesive organ^1,2^. This relatively small taxon currently has 10 valid species, which are considered strictly host-specific parasites of the spiral intestine of holocephalans (Chimaeriformes), mostly deep-sea dwelling cartilaginous fishes^3^. Gyrocotylideans are distributed worldwide and have been reported from 15 out of the 56 (25%) recognised extant species of holocephalans, with a high prevalence that may reach up to 100%^3,4^. To the best of our knowledge, each holocephalan that has been examined for endohelminths thus far hosts at least one gyrocotylidean species^5^. This suggests that major parts of gyrocotylidean diversity may be undiscovered. Recently, two new species of *Gyrocotyle*, from *Harriotta raleighana* Goode & Bean and *Hydrolagus mirabilis* (Collett), respectively, were described based only on genetic data, because the authors considered morphology to be totally unsuitable for species characterisation^6^.

To date, most of the species have been described from the North Atlantic^5–10^, whereas a few taxa have been reported from the southern seas^5,11–13^, including the type species *Gyrocotyle rugosa* Diesing, 1850. The only gyrocotylidean recorded in east Asian waters was found in *Chimaera phantasma* Jordan & Snyder from the coast of Japan and was identified as *Gyrocotyle fimbriata* Watson, 1911, in a brief report of Ichihara^14^ and in two unidentified species of *Hydrolagus* and *Harriotta* off Indonesia^3^. However, the former finding needs verification, because *G. fimbriata* was originally described as a parasite of *Hydrolagus colliei* (Lay & Bennett) from the west coast of the United States of America^15^.

In fact, reliable morphological identification is currently barely possible because of insufficient original descriptions dated mostly to the first half of the last century and overlapping diagnostic traits among the individual species^3,16^. Genetic identification is also limited, as sequence data are available, with one exception, for a few species from the North Atlantic, and some of them are most probably misidentified^6,17–20^. The reliable identification of many species is thus impossible and was often based only on their host and geographic origin.

However, some holocephalans may host more than a single gyrocotylidean species. *Chimaera monstrosa* L. may apparently be infected with *Gyrocotyle urna* (Grube & Wagener, 1852), *Gyrocotyle confusa* van der Land & Dienske, 1968, and *Gyrocotyle nybelini* (Fuhrmann, 1931). Another holocephalan, *Hydrolagus colliei*, may harbour *G*. *fimbriata* and *G. parvispinosa* van der Land & Dienske, 1968, and *Hydrolagus affinis* (de Brito Capello) was found to be the host of *Gyrocotyle major* van der Land & Templeman, 1968 and *Gyrocotyle abyssicola* van der Land & Templeman, 1968^9,10,21–23^. Additionally, Manter^24^ and Bandoni & Brooks^16^ questioned the strict host specificity of gyrocotylideans and reported *G. urna* and *G. fimbriata* as parasites of more than one holocephalan species.

Herein, we suggest an approach based on examination of voucher specimens of sequenced samples, i.e., hologenophores (see^25^). We document the unexpected genetic divergence of morphologically almost indistinguishable gyrocotylideans from the East China Sea and also provide the first detailed molecular and morphological characterisation of *Gyrocotyle rugosa* from the type host as a baseline for future studies.

## Results

### Molecular analysis of *Gyrocotyle* spp

In total, 17 specimens of *Gyrocotyle* spp. from Taiwan were genotyped and four distinct genotypes were identified based on partial sequences of the *COI*, *28S rRNA* and *18S rRNA* genes. The pairwise divergence (uncorrected p-value) within the particular genotypes (i.e., intragenotypic divergence) ranged from 0.2 to 0.8% for the *COI* dataset, whereas the sequences of *28S rDNA* were identical (the value was not calculated for *18S rRNA*, because a single sequence was obtained for each genotype). The intergenotypic pairwise divergences were 6.1‒9.1% for *COI*, 1.2‒3.9% for *28S rRNA,* and 0.9‒1.2% for *18S rRNA*. Considering isolates of *Gyrocotyle rugosa* from *Callorhinchus callorynchus* L. off Argentina, identical sequences of both *COI* and *28S rRNA* genes were obtained from two specimens sequenced. The pairwise divergence between them and possibly a conspecific isolate of *Gyrocotyle* sp. from *Callorhinchus milii* Bory de Saint-Vincent off Australia was 0.5% and 0.2% for the *28S rRNA* and *18S rRNA* genes, respectively.

For phylogenetic analysis, amphilinidean, caryophyllidean and spathebothriidean taxa were tested as potential outgroups. However, the numerous gaps in alignments caused a considerable loss of signal due to the removal of ambiguously aligned sequence positions. As rooted trees with isolates of none of the three tapeworm orders provided robust support for phylogenetic relationships among the gyrocotylidean taxa, their genetic relationships are herein depicted as an un-rooted network of two concatenated datasets, i.e. *28S rRNA* + *18S rRNA* genes and *COI* + *28S rRNA* + *18S rRNA* genes.

**Figure 1:**
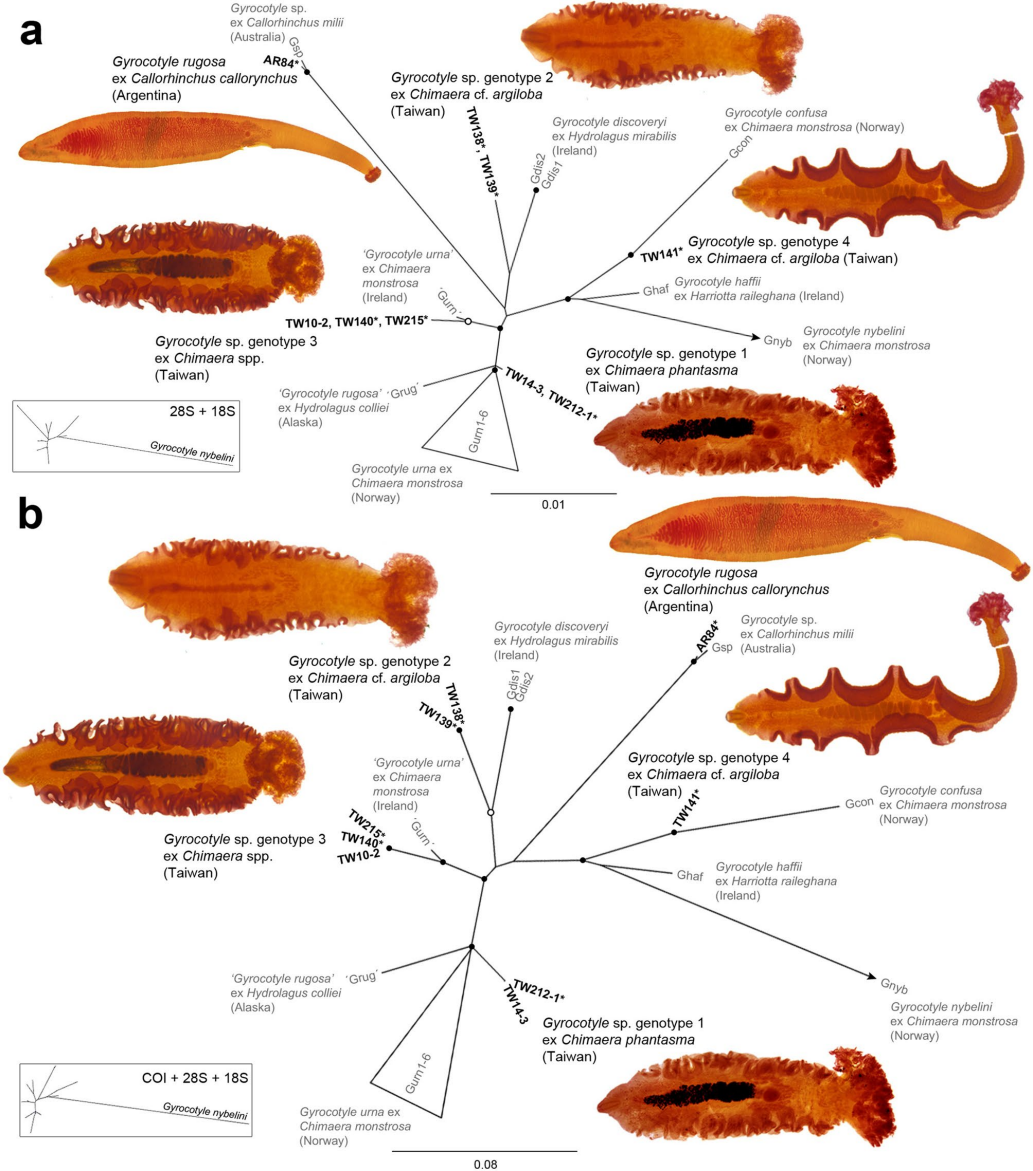
Estimation of genetic interrelationships among the isolates of the genus *Gyrocotyle.* Maximum likelihood analysis performed on two concatenated datasets of two (*28S rRNA* + *18S rRNA*) (**a**) and three (*COI* + *28S rRNA* + *18S rRNA*), (**b**) partial genes depicted as unrooted networks. Note the genetic diversity of the four genotypes of *Gyrocotyle* sp. from Taiwan, each of which clustered with *G. urna* off Norway + ‘*G. rugosa*’ off Alaska, *G. discovery* off Ireland, ‘*G. urna*’ off Ireland, and *G. confusa* off Norway, respectively, rather than together. Also note the cluster of *Gyrocotyle* sp. genotype 3 specimens isolated from *Chimaera phantasma* and *C.* cf. *argiloba*, respectively, and the close relatedness of *Gyrocotyle rugosa* from *Callorhinchus callorynchus* off Argentina with the isolate of *Gyrocotyle* sp. from *Callorhinchus milii* off Australia. Our original isolates are in black, with a micrograph of the morphological voucher; asterisks mark the hologenophores. The ultrafast bootstrap supports over 89% and 94% are shown as open circles and black dots, respectively. The full length of the branches and gene concatenations are in the rectangular box, the identification of isolates in Table 1.

Out of 17 specimens genotyped, only the specimens genetically unique in at least one genetic marker were chosen for the further analysis (for the complete list of the material examined, see Supplementary Table S1). Among the unique isolates, the four genotypes of *Gyrocotyle* off Taiwan did not cluster together but formed separate groups spread among other gyrocotylideans in both analyses (Fig. 1a,b). The individuals of *Gyrocotyle* sp. genotype 1 from *Chimaera phantasma* exhibited the highest genetic similarity with the specimens of *Gyrocotyle urna* from *Chimaera monstrosa* off Norway and an isolate identified as “*Gyrocotyle rugosa*” from *Hydrolagus colliei* in the Gulf of Alaska. Two specimens of *Gyrocotyle* sp. genotype 2 from *Chimaera* cf. *argiloba* diverged from the lineage with the isolates of *Gyrocotyle discoveryi* Bray, Waeschenbach, Littlewood, Harvolsen & Olson 2020 from *Hydrolagus mirabilis* off Ireland, and the specimens of *Gyrocotyle* sp. genotype 3 from *C. phantasma* and *C.* cf. *argiloba* grouped with the isolate identified as “*Gyrocotyle urna*” from *Chimaera monstrosa* off Ireland. The specimen of *Gyrocotyle* sp. genotype 4 appeared in the branch with the isolate of *Gyrocotyle confusa* from *Chimaera monstrosa* off Norway, both being closely related to *Gyrocotyle haffii* Bray, Waeschenbach, Littlewood, Harvolsen & Olson 2020 from *Harriotta raileghana* off the North Atlantic and *Gyrocotyle nybelini* from *Chimaera monstrosa* off Norway, which had by far the longest branch in both analyses (Fig. 1a,b).

Additionally, our specimen of *Gyrocotyle rugosa* off Argentina grouped with the only available isolate from the Southern hemisphere, *Gyrocotyle* sp. from *Callorhinchus milii* off Australia. These two specimens formed a relatively long branch, which was closely related to the group of *Gyrocotyle* sp. genotype 2 off Taiwan + *Gyrocotyle discoveryi* in the *28S rRNA* + *18S rRNA* gene network (Fig. 1a), though they exhibited a different topology when the *COI* gene was included (Fig. 1b). Moreover, the addition of the *COI* data increased the bootstrap support for some nodes when comparing with the dataset of ribosomal genes only.

### Morphological characterisation of specimens off Taiwan and Argentina

**Figure 2:**
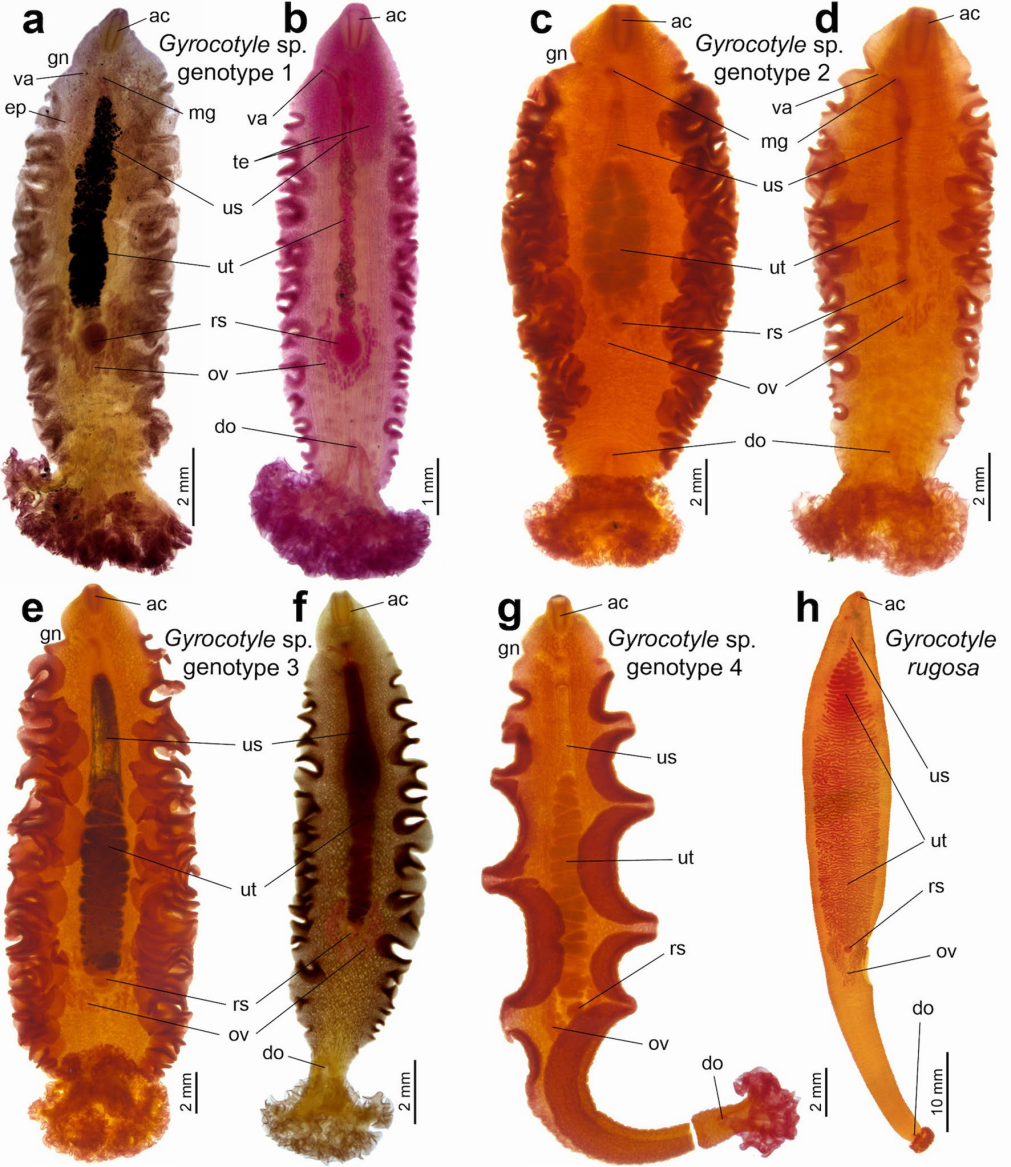
Whole mounts of *Gyrocotyle* spp. off Taiwan and Argentina. Morphological vouchers of *Gyrocotyle* sp. genotype 1 ex *Chimaera phantasma* (**a**), specimen TW212-1*; (**b**), specimen TW13-1, *Gyrocotyle* sp. genotype 2 ex *Chimaera* cf. *argiloba* (**c**), specimen TW138*; (**d**), specimen TW139*, *Gyrocotyle* sp. genotype 3 ex *C.* cf. *argiloba* (**e**), specimen TW215* and *C. phantasma* (**f**), specimen TW10-1, *Gyrocotyle* sp. genotype 4 ex *C.* cf. *argiloba* (**g**), specimen TW141*, all from Taiwan; and *Gyrocotyle rugosa* ex *Callorhinchus callorynchus* Argentina (**h**), specimen AR89*. Field numbers with asterisks above indicate the hologenophores. *ac* acetabulum, *do* dorsal opening, *ep* excretory pore, *gn* genital notch, *mg* male gonopore, *ov* ovary, *rs* receptaculum seminis, *te* testes, *us* uterine sac, *ut* uterus, *va* vagina.

**Figure 3:**
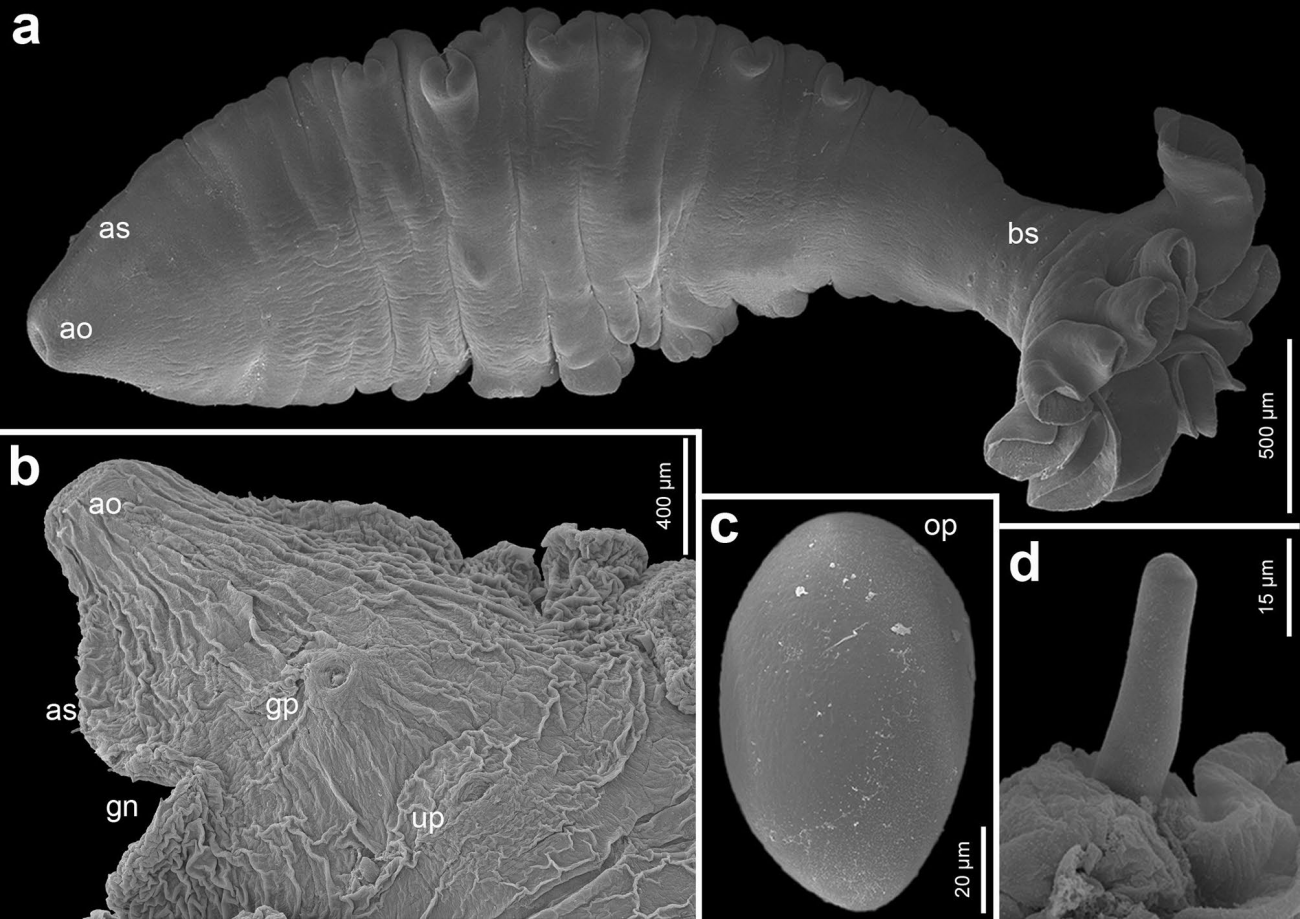
Scanning electron micrographs of *Gyrocotyle* sp. genotype 1 ex *Chimaera phantasma* (Taiwan). Ventral view of a juvenile specimen TW12-3; note the distribution of body spines restricted near the rosette, lateral plication, and weakly developed genital pores (**a**). The anterior body part of an adult TW12-3 specimen (**b**), intrauterine egg with smooth surface and shallow operculum (**c**), detail of an acetabular spine (**d**). *ao* acetabular opening, *as* acetabular spines, *bs* body spines, *gn* genital notch, *gp* genital papilla, *op* operculum, *up* uterine pore.

The specimens of *Gyrocotyle* sp. genotypes 1, 2 and 3 off Taiwan are similar in their general morphology, i.e., they possess heavily plicated lateral margins, a wide funnel and a large complex rosette. The copulatory papilla and external and internal seminal vesicles are present, and the male pore opens slightly posterior to the level of the vaginal pore. The ovary is U-shaped, the uterus coiled, and its distal part forms a well-developed uterine sac containing unembryonated eggs. Two excretory pores open posterolateral to the uterine pore (Figs. 2a‒f, 3a‒d).

However, these three genotypes can be distinguished from each other by the unique combination of morphological traits (Supplementary Table S2). Specimens of *Gyrocotyle* sp. genotype 1 possess a large acetabulum and a rosette of the same width or wider than the maximum body width. The acetabular spines are less numerous, and the dorsal body spines are small and distributed mostly on the posterior half of the body, whereas ventral spines are few and restricted to near the rosette. The testicular field reaches one-fourth of the uterus length, the ejaculatory duct is straight, and the uterine sac is middle-sized (Fig. 2a,b).

*Gyrocotyle* sp. genotype 2 is characterised by a large acetabulum and the rosette is not wider than the maximum body width. The acetabular spines are numerous and the body spines are small, with dorsal spines distributed on the posterior half of the body and near the lateral margins towards the acetabulum, whereas the ventral body spines are restricted to near the rosette; anterolateral spines (posterior to the acetabular spines) are also present. The testicular field reaches one-half of the uterus, the ejaculatory duct is coiled, and the uterine sac is middle-sized (Fig. 2c,d).

*Gyrocotyle* sp. genotype 3 possesses a small acetabulum and the rosette is not wider than the maximum body width. The acetabular spines are less numerous, body spines are large and distributed over the whole body surface. They are more numerous on the dorsal than on the ventral side, and their density is highest on the funnel, decreasing towards the acetabulum. The testicular field reaches one fourth of the uterus, the ejaculatory duct is straight, and the uterine sac is large (Fig. 2e,f).

The single specimen of *Gyrocotyle* sp. genotype 4 markedly differs from the specimens of all the genotypes described above by its lateral margins with few deep folds, numerous acetabular spines, a relatively narrow funnel and rosette, and the ovary being far from both the dorsal opening and the posterior body margin (Fig. 2g).

*Gyrocotyle rugosa* from *Callorhinchus callorynchus* off Argentina is typified by possessing an elongated body with crenulate lateral margins, a small rosette, a weakly developed uterine sac, a branched uterus, a V-shaped ovary and embryonated intrauterine eggs (Fig. 2h).

### Molecular identification of *Chimaera* spp. off Taiwan

**Figure 4:**
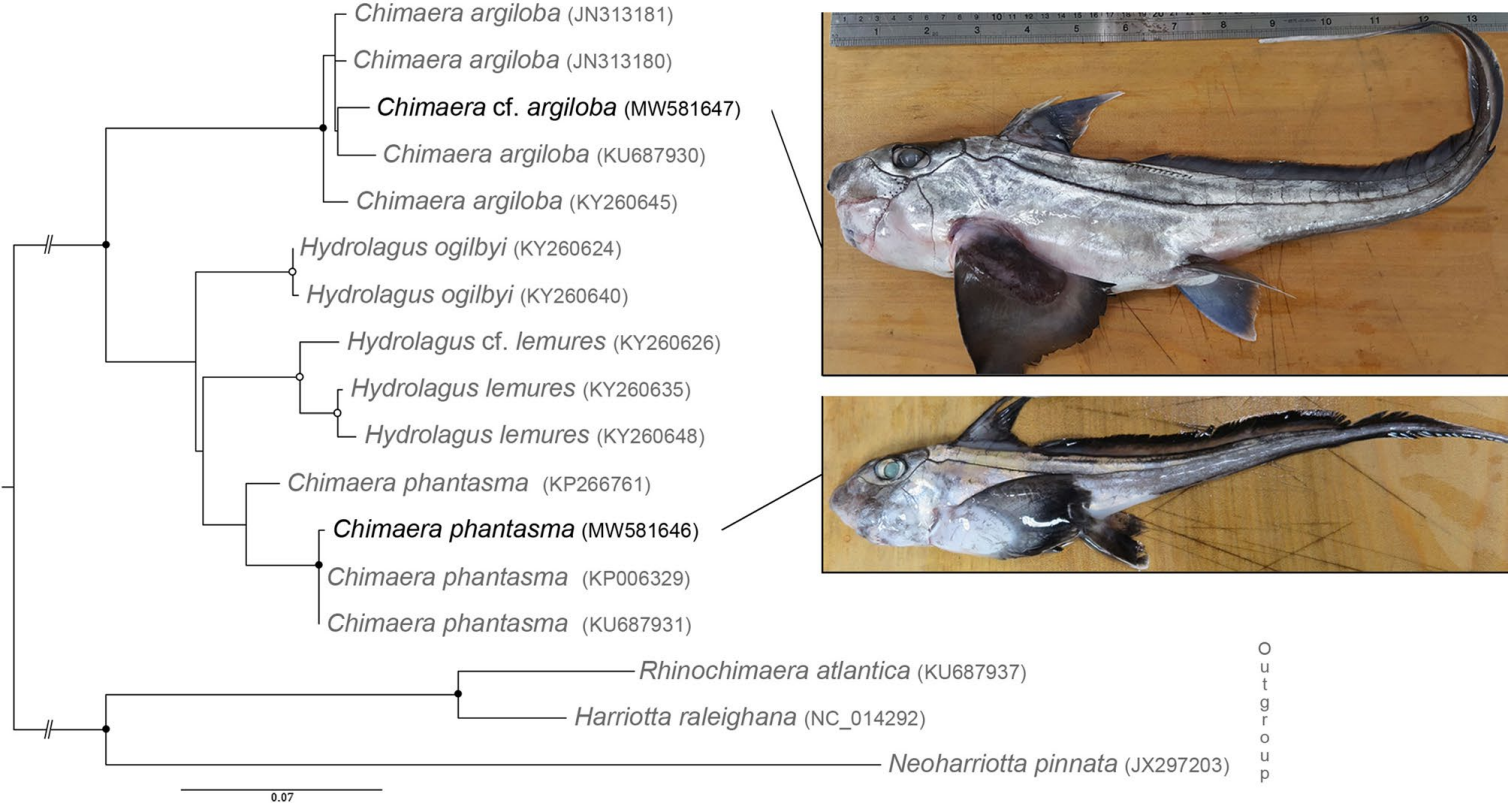
Hosts of gyrocotylideans off Taiwan and their phylogenetic positions. Maximum likelihood analysis on a partial *COI* gene dataset distinguished two host genotypes, i.e., *Chimaera phantasma* and *C.* cf. *argiloba*. Newly generated sequences are in black. The ultrafast bootstrap supports over 89% and 94% are depicted as open circles and black dots, respectively. The scale bar represents the number of substitutions. Numbers in parentheses are accession numbers in the GenBank database.

The analysis of the hosts’ *COI* gene distinguished two host genotypes (Fig. 4). The first genotype clustered within the *Chimaera phantasma* clade, whereas the second one nested with the isolates of *Chimaera argiloba* Last, White & Pogonoski off Australia, Indonesia and New Caledonia. Although the identification of the second Taiwanese genotype as *Chimaera* cf. *argiloba* is provisional due to its distinct geographical origin, it is evident that we examined two different host species from the East China Sea.

## Discussion

Almost 50 years ago, Simmons^26^ called gyrocotylideans a “century-old enigma” and this status still persists despite the advent of more advanced identification methods^3^. The poor understanding of the group (e.g., the complete life cycle of none of the species is known) is linked with the scarcity of available data and the biological peculiarities of these tapeworms and their holocephalan hosts. In particular, most of the host species are rarely available deep-sea dwellers, which often could not be examined fresh or were frozen with their parasites prior to examination. If isolated alive, gyrocotylideans exhibit an unusual morphological variability due to the contraction of their large bodies and as a result of different fixative procedures which were tested to ensure their relaxation (e.g.^27^). Despite these issues, several comprehensive studies have been conducted, e.g.^15,16,21,28^, which provided deep insight into the biology, ecology and taxonomy of these enigmatic tapeworms. Nevertheless, the poor quality of the specimens studied and the use of different, not always appropriate, methods of parasite fixation, unintentionally affected the quality of morphological descriptions of most gyrocotylidean species, which prevented the establishing of clear morphological borders to delimit individual species. As a result, the informative value of morphological traits used for species delimitation should be re-assessed, based on the simultaneous use of molecular data, i.e., the use of hologenophores to match morphology and molecular data. Existing problems with species delimitation and morphological variability even led to complete omission of morphological characterisation of two new species described just recently^6^.

Herein, the genotyping of the *Gyrocotyle* spp. specimens acquired in Taiwan revealed four distinct genotypes, each one more related to the North Atlantic isolates identified as “*Gyrocotyle urna*” off Ireland (the isolate is genetically diverse from *G. urna* off Norway), “*G. rugosa*” off Alaska (probably misidentified, see below), *G. discoveryi* off Ireland and *G. confusa* off Norway, respectively, than to each other*.*

In addition to casting doubts on the restriction of gyrocotylideans to individual oceans, our data also question the proclaimed strict host specificity^3,7^, because specimens of *Gyrocotyle* sp. genotype 3 were found in two hosts species, which are not the closest relatives to one another—*C. phantasma* and *C.* cf. *argiloba* (Fig. 4). Broader host specificity was also reported for *G. fimbriata,* which was found in *Hydrolagus colliei* and *Chimaera phantasma*, and for *G. rugosa*, recorded in *Callorhinchus callorynchus* and *C. milii*^14,15,24,29^*. Gyrocotyle urna* was also found in several holocephalans, including *Chimaera monstrosa*, *Callorhinchus callorynchus*, *Hydrolagus ogilbyi* Waite and *H. colliei*^24,29,30^. In contrast, Bandoni & Brooks^16^ revised the host spectrum of this parasite, considering *C. monstrosa* as the only host of *G. urna*.

The suitability of the molecular markers employed for this group also requires attention, because a considerable amount of phylogenetic information was also lost in the un-rooted dataset due to treatment of the numerous gaps in the *28S rRNA* alignment. The involvement of partial *COI* gene sequences seemed to be informative for estimating gyrocotylidean phylogeny, because we obtained a no-gap *COI* alignment and improved support for some nodes in the three-gene network. The suitability of this marker requires assessment employing further taxa, because except for our isolates off Taiwan and Argentina, only a single sequence of the *COI* gene (i.e., that of *G. urna* off Norway; GenBank acc. no. JQ268546) is currently available.

A single specimen of *Gyrocotyle* sp. genotype 4 was conspicuously different morphologically from the remaining ones by having few folds on the lateral margins, many acetabular spines, a narrow funnel and a small rosette. However, its formal description as a new species would be premature, because only a single specimen was found. Morphological differences among the specimens of the other genotypes were not so obvious, even though a careful examination of the hologenophores allowed us to find several morphological traits that were characteristic for particular genotypes (see “Results” section). Among them, the number of acetabular spines and the distribution of the body spines and their size may be potentially useful for species differentiation, especially because the body contraction can hardly affect them. Since body contraction cannot be absolutely excluded even when live specimens are properly fixed, its effect could be overcome to some degree by an evaluation of ratios related to the main body dimensions (e.g., length of uterine sac/total body length) rather than comparison of total measurements of internal structures.

The specimens off Taiwan most probably represent several new species, but we decided not to describe them formally as new taxa, mainly because of the shortage of comparative data. In addition to these specimens, two hologenophores of *Gyrocotyle rugosa* off Argentina were examined, which made it possible to characterise the type species of the genus. The host of *G. rugosa* described by Diesing^10^ was questionable until *Callorhynchus antarcticus* (= *C. callorynchus*—see^31^) off New Zealand was finally established as its currently accepted type host^3,32^. *Gyrocotyle rugosa* was found in coastal waters of South America, South Africa and New Zealand as a parasite of *C. callorynchus* and *C. milii*, suggesting its broader host specificity^16,24^. Our specimens from *C. callorynchus* off Argentina were identified as *G. rugosa* based on crenulated (i.e., without any folds) lateral margins, a tiny uterine sac, a branched uterus and embryonated eggs in the uterine sac; the latter two traits are unique to this species^21^. Genetically, it clustered with an unspecified isolate of *Gyrocotyle* from *C. milii* off Australia, and these specimens seem to be conspecific.

In contrast, an isolate from *Hydrolagus colliei* off Alaska identified as *G. rugosa* (GenBank acc. nos. AF286925 and AF124455) was apparently misidentified, because (i) it was found in an unrelated definitive host (*H. colliei* belongs to the family Chimaeridae, whereas the type host to the family Callorhinchidae), (ii) its distant geographic origin (the type locality of *G. rugosa* is unclear, but it is definitely in the Southern hemisphere), and (iii) its genetic divergence from our isolate of *G. rugosa* from the type host off Argentina. The isolate from *H. colliei* may represent *Gyrocotyle fimbriata* or *G. parvispinosa*, which have been reported from this host off the Pacific coast of North America, but its identification was not possible because morphological vouchers were not available to the present authors.

Gyrocotylideans were generally considered to be oioxenous, i.e. strictly specific parasites sensu Euzet and Combes^33^, with each gyrocotylidean species parasitising a single holocephalan species. Although several species were reported from two or more hosts species^16,24^, these findings are usually considered as misidentifications due to the unclear taxonomy of the order. Moreover, some holocephalans, such as *Ch. monstrosa*, *H. colliei*, *H. affinis*, and *Ca. callorynchus*, were often found to harbour two or more gyrocotylidean species, one common and the other rare^9,10,21–23^. Our findings of *Gyrocotyle* sp. genotypes 1 and 3 in *Ch. phantasma* and *Gyrocotyle* sp. genotypes 2, 3 and 4 in *Ch.* cf. *argiloba* suggested stenoxenous host specificity (i.e., the occurrence in a few closely related hosts) of gyrocotylideans, because the specimens of genotype 3 were found in both species of *Chimaera*. The obvious genetic similarity of our *G. rugosa* specimen from *Ca. callorynchus* and the isolate of *Gyrocotyle* sp. from *Ca. milii* also questions the strict specificity of this group, but morphological vouchers of the latter, which are necessary for the confirmation of their conspecificity, are not available.

Our genetic analyses provided insight into the interrelationships among the gyrocotylideans, even though the absence of a suitable outgroup did not enable us to broadly assess the possible evolutionary scenario of this earliest evolving group of tapeworms. Moreover, genetic data on only half of the nominal species of *Gyrocotyle* are available, not considering the possibility of misidentifications of previously sequenced specimens, for which hologenophores are not available. However, some clues of host-parasite coevolution can be inferred from the network. The mutual genetic distance of species/genotypes from the same host species suggests multiple colonisation events rather than co-speciation with their hosts within the order. It seems that *G. phantasma* might have been colonised by *Gyrocotyle* sp. genotype 1 or genotype 3, because these two genotypes are not the closest relatives in our analyses. The same pattern is obvious for *C.* cf. *argiloba* parasitised by *Gyrocotyle* sp. genotype 2, 3 and 4, and also for *C. monstrosa*, which harbours *G. urna, G. confusa* and *G. nybelini.* Indeed, Colin et al.^27^ considered these species from *C. monstrosa* to be conspecific, but our genetic data support the validity of three separate and genetically distant species. Moreover, *G. nybelini* formed by far the most distant lineage among all isolates, which may suggest the validity of the genus *Gyrocotyloides* Furhmann, 1931.

Genetic divergence of congeneric tapeworms from the same host species was also observed in several elasmobranch/teleost-cestode assemblages, e.g., *Acanthobothrium* spp. (Onchoproteocephalidea) and the mumburarr whipray *Urogymnus acanthobothrium* Last, White & Kyne; *Echeneibothrium* spp. (Rhinebothriidea) and the yellownose skate *Dipturus chilensis* (Guichenot); and *Pseudoendorchis* spp. (Onchoproteocephalidea) and the catfish *Pimelodus maculatus* Lacepède^34–36^.

The aim of this paper was to provide new insight into the phylogenetic relationships within the enigmatic order Gyrocotylidea, but, in particular, to demonstrate the lack of geographical patterns in the distribution of most its species and the limited suitability of current morphological characteristics for species circumscription. Herein, we have outlined a methodology (fixation of live specimens with hot fixative and the exclusive use of hologenophores) that should be used in future taxonomic, ecological and biogeographical studies of gyrocotylideans in order to reliably circumscribe their actual species diversity and to unravel associations with their hosts, a relict group of marine vertebrates. Gyrocotylideans represent one of the key groups of parasitic flatworms (Neodermata) in terms of a better understanding of their evolutionary history and the switch of free-living flatworms to parasitism.

## Material and methods

Fifteen individuals of *Chimaera* spp. (Chimaeridae) from the fish market in Yilan City, northern Taiwan, and several individuals of *Callorhinchus callorynchus* (Callorhinchidae) off Mar del Plata, Argentina, were examined from 2004 to 2019. Their morphological identification followed Ebert et al.^37^; isolation of their genomic DNA and amplification of partial mitochondrial *cytochrome c oxidase subunit 1* (*COI*) genes were performed according to Holmes et al.^38^. Purification of the amplicons, sequencing method and processing of raw sequence data were same as for the parasites (see below).

Live tapeworms isolated from the spiral valve of the above-mentioned holocephalans (prevalence 100%) were processed using standard helminthological techniques (see^39^); specimens were fixed with almost boiling water and then placed in 70% non-denaturated ethanol. This approach allowed the isolation of DNA from a small piece of the parasite tissue, whereas the rest of the body was used for morphological observations as a hologenophore (see^25^). Additionally, paragenophores (an individual putatively conspecific with a hologenophore) were included in our analyses, because no mixed infections, i.e., two different parasite species (genotypes in our case) in a single host individual were recorded. The vouchers were deposited in the Helminthological collection of the Institute of Parasitology, Biology Centre of the Czech Academy of Sciences, České Budějovice, Czech Republic (IPCAS C-869–872).

Isolation of genomic DNA and amplification of two nuclear genes (*18S rRNA* and *28S rRNA*) followed Olson et al.^40^. The partial *COI* gene was amplified by newly designed GcotCO1F (5′-ACTTTAGATCATAAGCGTATTGG-3′) and GcotCO1R1 (5′-AGCATAGTTATACCAGCAGC-3′) primers (courtesy of H. Hansen, Norwegian Veterinary Institute, Oslo, Norway); PCR was initiated by denaturation at 95 °C for 5 min, followed by 35 cycles of 95 °C for 1 min, 50 °C for 1 min, and 72 °C for 90 s, and terminated at 72 °C for 5 min. The amplicons were enzymatically purified^41^ and Sanger sequenced using PCR primers; nuclear genes were also sequenced using internal primers^40,42^. Contiguous sequences were aligned and inspected for ambiguous bases using Geneious^43^. In total, 26 original sequences of gyrocotylideans and *Chimaera* spp. were submitted to the GenBank database (accession numbers in Table 1).

**Table 1:** List of isolates with GenBank accession numbers of *18S rRNA, 28S rRNA* and *cytochrome c oxidase subunit 1* (*COI*) genes of *Gyrocotyle* spp. and *COI gene* of *Chimaera* spp. used in genetic analyses.

Species	Coll. nos	Host	Locality	Acc. no. 18S	Acc. no. 28S	Acc. no. *COI*	References
*Gyrocotyle urna*	Gurn1	*Chimaera monstrosa*	Unknown, Norway	–	AJ228799	–	^49^
*Gyrocotyle urna*	Gurn2	*Chimaera monstrosa*	Fjord near Bergen, Norway	AJ228782	AF286924	–	^17^
*Gyrocotyle urna*	Gurn3	*Chimaera monstrosa*	Fjord near Bergen, Norway	–	AY157178	JQ268546	^19,20^
*Gyrocotyle urna*	Gurn4	*Chimaera monstrosa*	Coast of Finnmark, Norway	–	MN657012	–	^6^
*Gyrocotyle urna*	Gurn5	*Chimaera monstrosa*	Coast of Troms, Norway	MN655884	MN657013	–	^6^
*Gyrocotyle urna*	Gurn6	*Chimaera monstrosa*	Unknown fjord, Norway	MN655883	MN657015	–	^6^
*'Gyrocotyle urna'*	'Gurn´	*Chimaera monstrosa*	Goban Spur, Ireland	MN655882	MN657010	–	^6^
*Gyrocotyle confusa*	Gcon	*Chimaera monstrosa*	Coast of Finnmark, Norway	–	MN657014	–	^6^
*Gyrocotyle nybelini*	Gnyb	*Chimaera monstrosa*	Coast of Finnmark, Norway	MN655885	MN657016	–	^6^
*Gyrocotyle discoveryi*	Gdis1	*Hydrolagus mirabilis*	Goban Spur, Ireland	MN655879	MN657003	–	^6^
*Gyrocotyle discoveryi*	Gdis2	*Hydrolagus mirabilis*	Porcupine Bight, Ireland	MN655881	MN657007	–	^6^
*Gyrocotyle haffii*	Ghaf	*Harriotta raileghana*	Goban Spur, Ireland	MN655880	MN657006	–	^6^
*Gyrocotyle rugosa*	´Grug´	*Hydrolagus colliei*	Gulf of Alaska, USA	AF124455	AF286925	–	^50^
*Gyrocotyle* sp.	Gsp	*Callorhinchus milii*	Tasman Sea, Australia	EU343741	EU343735	–	^50^
*Gyrocotyle* sp. gen. 1	TW212-1	*Chimaera phantasma*	East China Sea, Taiwan	MW587254	MW587259	MW581648	This study
*Gyrocotyle* sp. gen. 1	TW14-3	*Chimaera phantasma*	East China Sea, Taiwan	–	MW587260	MW581649	This study
*Gyrocotyle* sp. gen. 2	TW138	*Chimaera* cf. *argiloba*	East China Sea, Taiwan	MW587255	MW587261	MW581650	This study
*Gyrocotyle* sp. gen. 2	TW139	*Chimaera* cf. *argiloba*	East China Sea, Taiwan	–	MW587262	MW581651	This study
*Gyrocotyle* sp. gen. 3	TW10-2	*Chimaera phantasma*	East China Sea, Taiwan	–	MW587263	MW581652	This study
*Gyrocotyle* sp. gen. 3	TW140	*Chimaera* cf. *argiloba*	East China Sea, Taiwan	–	MW587264	MW581653	This study
*Gyrocotyle* sp. gen. 3	TW215	*Chimaera phantasma*	East China Sea, Taiwan	MW587256	MW587266	MW581654	This study
*Gyrocotyle* sp. gen. 4	TW141	*Chimaera* cf. *argiloba*	East China Sea, Taiwan	MW587257	MW587265	MW581655	This study
*Gyrocotyle rugosa*	AR84	*Callorhinchus callorynchus*	Coast near Mar del Plata, Argentina	MW587258	MW587267	MW581656	This study
*Chimaera phantasma*	TW12h	–	East China Sea, Taiwan	–	–	MW581646	This study
*Chimaera* cf. *argiloba*	TW14h	–	East China Sea, Taiwan	–	–	MW581647	This study

Phylogenetic analyses were computed based on two concatenated datasets of our original sequences and those available from GenBank. The sequences of three genes were separately aligned by E-INS-I (*18S rRNA* and *28S rRNA*) and L-INS-I (*COI*) algorithms in MAFFT ver.7.388^44^; the alignments were inspected by eye in Geneious and doubtful positions were removed. Substitution models for *18S rRNA* (GTR + F + I), *28S rRNA* (TVM + F + I + G4) and *COI* (TIM3 + F + I, HKY + F, and K3Pu + F + G4 for the 1st, 2nd, and 3rd codon positions, respectively) gene alignments were estimated under AICc criterion with an implemented test of model violation in ModelFinder using IQ-Tree ver. 2.0.5^45–47^. IQ-Tree was also employed in the construction of phylogenetic trees under the Maximum Likelihood method with ultrafast bootstrapping (1000 replicates)^48^. These methods were also applied for host molecular identification employing TIM3e + G4, F81 + F, and TIM2 + F + G4 substitution models for the 1st, 2nd, and 3rd *COI* codon positions, respectively.

## Supplementary Information

Supplementary Information.

## Data Availability

The datasets generated during the current study are available from the corresponding author upon request.
