# Supplementary material for: Hidden diversity of the most basal tapeworms (Cestoda, Gyrocotylidea), the enigmatic parasites of holocephalans (Chimaeriformes)
Source: Sci Rep. 2021 Mar 9;11:5492. doi: 10.1038/s41598-021-84613-y (PMC7970904; doi:10.1038/s41598-021-84613-y)
Supplement: Supplementary file 1 — Supplementary Information. [file 41598_2021_84613_MOESM1_ESM.pdf]

Hidden diversity of the most basal tapeworms (Cestoda, Gyrocotylidea), the enigmatic parasites of holocephalans (Chimaeriformes)

Daniel Barčák, Chia-Kwung Fan, Pasaikou Sonko, Roman Kuchta, Tomáš Scholz, Martina Orosová, Hsuan-Wien Chen, Mikuláš Oros

Supplementary table S1. Morphological vouchers examined in this study; paragenophores (without asterisk) ranked to genotypes based on their morphological similarity with hologenophores.

Gyrocotyle sp. genotype 1
Material examined: coll. nos. TW12-3†, TW13-1, TW13-2*, TW14-1, TW212-1*, TW212-2*, TW213-2†*, TW214-1*, TW214-2* ex *Chimaera phantasma*
Locality: Yilan, Taiwan, East China Sea

Gyrocotyle sp. genotype 2
Material examined: coll. nos. TW138*, TW139* ex *Chimaera* cf. *argiloba*
Locality: Yilan, Taiwan, East China Sea

Gyrocotyle sp. genotype 3
Material examined: coll. nos. TW10-1, TW215* ex *Chimaera phantasma*; TW140* ex *Chimaera* cf. *argiloba*
Locality: Yilan, Taiwan, East China Sea

Gyrocotyle sp. genotype 4
Locality: Yilan, Taiwan, East China Sea
Material examined: TW141* ex *Chimaera* cf. *argiloba*

Gyrocotyle rugosa Diesing, 1850 – type species
Material examined: AR84*, AR89* ex *Callorhinchus callorynchus*
Locality: Mar del Plata, Argentina, South-West Atlantic Ocean

* hologenophores
† specimens examined by scanning electron microscope

Supplementary table S2. Morphometric analysis of *Gyrocotyle* spp. from Taiwan and Argentina. Measurements in micrometers unless otherwise stated, number of measurements in parentheses.

Specimen	***Gyrocotyle* sp. genotype 1, Taiwan (n=6)**	***Gyrocotyle* sp. genotype 2, Taiwan (n=2)**	***Gyrocotyle* sp. genotype 3, Taiwan (n=3)**	***Gyrocotyle* sp. genotype 4, Taiwan (n=1)**	***Gyrocotyle rugosa*, Argentina (n=2)**

Host	*Chimaera phantasma*	*Chimaera* cf. *argiloba*	*Chimaera phantasma* & *Chimaera* cf. *argiloba*	*Chimaera* cf. *argiloba*	*Callorhinchus callorynchus*
Body: length (mm)	8.6–17.2	17.1; 20.9	12.0–28.6	29	71
max. width (mm)	2.5–5.6	5.7; 8.6	4.4, 5.4	7.2	10.6; 10.9
width / length (%)	25–33	33; 41	24, 45	25	15
Genital notch	Weak	Conspicuous	Weak or Conspicuous	Conspicuous	Narrow
distance from AM (mm)	1.2–1.6	2.4; 2.8	2.1–2.9	2.8	4.1
Lateral margin	Heavily plicate	Heavily plicate	Heavily plicate	Plicate	Crenulate
type of folds	Simple	Simple	Simple	Simple	NA
maximum depth of folds (mm)	0.4–0.9	0.8	0.7	1.6	NA
width of folds (mm)	0.8–2.0	1.9; 2.5	1.2–3.7	3.3	NA
number of folds per side	17–22	More than 18	More than 17	4	NA
Funnel[1]: width (narrowest)	1,153–2,494	2,278; 2,591	1,168–2,923	1,245	2,458; 2,284
funnel width / body width (%)	44–51	40; 30	27, 35	17	23; 21
terminal sphincter	No	No	No	No	No
Rosette[2]: type	Complex	Complex	Complex	Complex	Complex
width (mm)	3.3–5.6	5.4; 5.8	4.1–7.0	3.7	4.5; 3.6
rosette width / body width (%)	94–131	94; 67	77, 96	52	42; 33
Acetabulum (AC): size	793–1,124 × 400–528	1,775 × 1,105;<br>1,786 × 1,334	1,143–1,447 × 713–932	1,391 × 814	1,745 × 1,477;<br>2,094 × 1,166
AC length / AM-UP distance (%)	48–56	45; 55	34–47	37	35; 40
Acetabular spines: number per side	12–22	21–29	18–22	52	NA
size	(30) 112–154 × 16–24	(8) 145–202 × 14–26	(9) 173–254 × 16–30	(4) 140–162 × 11–15	c. 230 × NA
Body spines: distribution	DS: posterior half<br>VS: near rosette	DS: posterior body half & lateral rows up to AC;<br>VS: near rosette & anterolateral spines	Whole body, more numerous on DS	DS: posterior half;<br>VS: near rosette	DS: near rosette<br>VS: not observed
size	(8) 109–150 × 22–45	(10) 107–123 × 24–35	(11) 121–327× 25–61	(9) 201–297 × 39–56	NA
type[3]	“urna-type”	“urna-type”	“urna-type”	“urna-type”	“urna-type”
Testes: size	(18) 59–104 × 48–100	(4) 64–73 × 58–68	(8) 53–103 × 51–88	(5) 86–93 × 67–84	(7) 82–94 × 65–83
approximate number	Hundreds	Hundreds	Hundreds	Hundreds	Hundreds
last testis from AM (mm)	2.9–4.8	5.6	9.2	8,5; 8,6	12.9
position of last testis	First quarter of UT	Middle part of UT	First quarter of UT	First quarter of UT	First quarter of UT
Testicular fields: lengths	2.0–4.2	4.7; 5.1	4.0–8.0	7.0; 7.2	12.5
length / body length (%)	21–27	27, 30	22–28	24, 25	18
External & internal seminal vesicle	Both present	Both present	Both present	Both present	Both present

Ejaculatory duct	Straight	Coiled	Straight	Straight	Straight
Copulatory papilla	Present	Present	Present	Present	Present
Uterus (UT): type	Tubular, coiled	Tubular, coiled	Tubular, coiled	Tubular, coiled	Branched
size	3,221–4,763 × 305–1,947	3,529 × 515; 5,760 × 2,512	2,605–9,952 × 889–2,489	9,298 × 1,502	44.1× 8.8 mm; 37.1 × 8.4 mm
uterus length / body length (%)	24–37	21, 28	22–33	32	62, 52
uterus width / body width (%)	12–40	9, 29	20, 27	21	83, 77
Uterine sac (US): size	829–2,160 × 140–1,370	1,348 × 393; 2,788 × 1,387	2,288–6,167 × 1,075–1,679	4,118 × 839	767 × 341; 920 × 375
US length / uterus length (%)	26–53	24, 48	62–88	44	2, 3
US length / body length (%)	8–18	8, 13	19–22	14	1
Ovary: shape	U	U	U	U	V
size	1,477–3,389 × 860–2,428	3,121 × 2,607; 2,679 × 2,989	2,368–3,395 × 1,916	2,939 × 2,642	6,064 × 4,130; 7,112 × 5,117
distance from PM (mm) (A)	3.4–4.7	7.1, 7.9	6.2–7.3	13.7	18.3, 22.4
A value / body length (%)	25–33	42; 38	26; 34	47	26, 32
Vitelline follicles: size	(17) 16–43 × 15–37	(17) 30–55 × 26–42	(14) 21–66 × 18–55	(6) 40–56 × 37–50	(11) 39–64 × 30–56
Vagina: muscular part	Straight	Straight	Straight	Straight	Sinuous
Seminal receptacle: size	406–796 × 451–622	585 × 464; 442 × 549	611 × 850; 440 × 369	654 × 813	1,188 × 1,205; 1,013 × 1,122
Excretory pores from AM (mm) (B)	1.8–3.4	4.2, 5.8	2.7–6.9	7.1, 7.7	6.6–9.5
B value / body length	17–29	25, 28	15–28	18, 20	22–25
Vaginal pore position	Anterior to MP	Anterior to MP	Anterior to MP	Anterior to MP	Anterior to MP
Uterine pore (UP) from AM	1,523–2,226	3,203; 3,962	2,438–4,268	3,770	5,040; 5,280
MP-UP / AC-UP (%)	37–55	71, 69	46–59	53	69, 59
Eggs in uterine sac	Unembryonated	Unembryonated	Unembryonated	Unembryonated	Embryonated
size	(6) 73–92 × 46–63	(8) 71–94 × 49–66	(6) 87–94 × 55–70	(4) 84–91 × 57–60	(6) 72–82 × 56–68

Abbreviations: AC - acetabulum, AM - anterior margin, DS - dorsal side, MP - male pore, PM - posterior margin, UP - uterine pore, US - uterine sac, UT - uterus, VS - ventral side.

[1] the narrowest part of the body posterior to last lateral folds and near the rosette

[2] the most posterior part of the body usually with numerous frills

[3]see Land & Dienske 1968
